# Deep Roots for Aboriginal Australian Y Chromosomes

**DOI:** 10.1016/j.cub.2016.01.028

**Published:** 2016-03-21

**Authors:** Anders Bergström, Nano Nagle, Yuan Chen, Shane McCarthy, Martin O. Pollard, Qasim Ayub, Stephen Wilcox, Leah Wilcox, Roland A.H. van Oorschot, Peter McAllister, Lesley Williams, Yali Xue, R. John Mitchell, Chris Tyler-Smith

**Affiliations:** 1 The Wellcome Trust Sanger Institute, Wellcome Genome Campus, Hinxton, Cambridgeshire CB10 1SA, UK; 2 Department of Biochemistry and Genetics, La Trobe Institute of Molecular Sciences, La Trobe University, Melbourne, VIC 3086, Australia; 3 Department of Medicine, University of Cambridge, Cambridge CB2 2QQ, UK; 4 Australian Genome Research Facility, Melbourne, Victoria 3052, Australia; 5 Division of Systems Biology and Personalised Medicine, Walter and Eliza Hall Institute of Medical Research, Melbourne, VIC 3052 Australia; 6 Office of the Chief Forensic Scientist, Victorian Police Forensic Services Department, Melbourne, VIC 3085, Australia; 7 Griffith University, Brisbane, QLD 4222, Australia; 8 Community Elder and Cultural Advisor, Brisbane, QLD 4011, Australia

## Abstract

Australia was one of the earliest regions outside Africa to be colonized by fully modern humans, with archaeological evidence for human presence by 47,000 years ago (47 kya) widely accepted [1, 2]. However, the extent of subsequent human entry before the European colonial age is less clear. The dingo reached Australia about 4 kya, indirectly implying human contact, which some have linked to changes in language and stone tool technology to suggest substantial cultural changes at the same time [3]. Genetic data of two kinds have been proposed to support gene flow from the Indian subcontinent to Australia at this time, as well: first, signs of South Asian admixture in Aboriginal Australian genomes have been reported on the basis of genome-wide SNP data [4]; and second, a Y chromosome lineage designated haplogroup C^∗^, present in both India and Australia, was estimated to have a most recent common ancestor around 5 kya and to have entered Australia from India [5]. Here, we sequence 13 Aboriginal Australian Y chromosomes to re-investigate their divergence times from Y chromosomes in other continents, including a comparison of Aboriginal Australian and South Asian haplogroup C chromosomes. We find divergence times dating back to ∼50 kya, thus excluding the Y chromosome as providing evidence for recent gene flow from India into Australia.

## Highlights

• We have sequenced 13 Aboriginal Australian Y chromosomes
• These diverged from Y chromosomes in other continents around 50,000 years ago
• They diverged from Papua New Guinean Y chromosomes soon after this
• We find no evidence for Holocene male gene flow to Australia from South Asia

## Graphical Abstract

**Figure:**
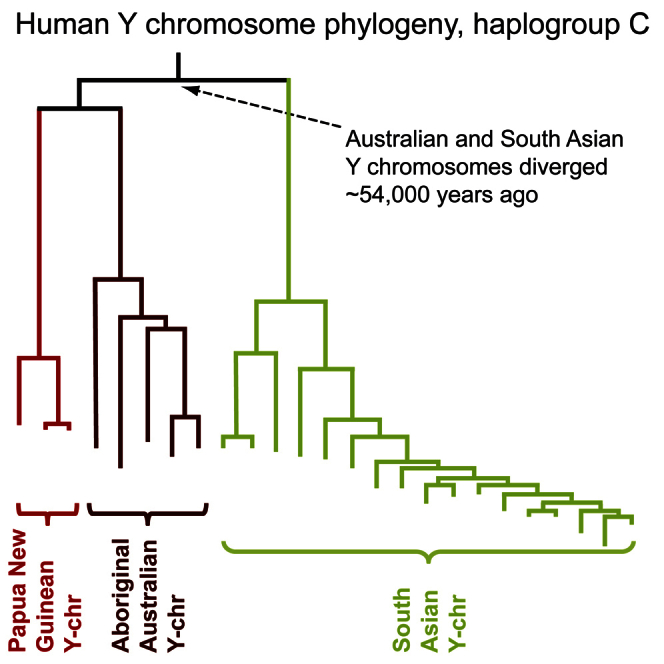

## Teaser

Bergström et al. show that Aboriginal Australian Y chromosomes diverged from Eurasian, including South Asian, Y chromosomes ∼50,000 years ago. This is around the time that Australia was first populated and thus disproves the previous hypothesis of prehistoric Y chromosome gene flow from India ∼5,000 years ago.

## Results and Discussion

### Genotyping and Sequencing of Aboriginal Australian Y Chromosomes

144 self-identified Aboriginal Australian males who volunteered to participate in the Genographic Project were previously typed with Y SNPs to assign them to major haplogroups [6]. A large fraction (∼70%) of Aboriginal Australian males today carry Y chromosomes of Eurasian origin (∼59% European) due to admixture in the last ∼200 years after the European colonization of Australia [7]. Among the individuals with indigenous Y chromosomes, 44% belong to haplogroup C, with 42% being C-M347 and 2% the basal C-M130^∗^. Paragroup K^∗^ constitutes 56% of indigenous Y chromosomes, with 27% being S-P308, 2% being haplogroup M-M186, and 27% being the basal K-M526^∗^ [6]. Although we note that other nomenclatures with relevance to these haplogroups exist [8] or could be proposed, these labels suffice for the purposes of our present study, and for simplicity we hereafter refer to C-M347 and C-M130^∗^ as Aboriginal Australian C, to S-P308 and K-M526 as K^∗^, and to M-M186 as M. For distinguishing subclades of haplogroup C, we also make use of the haplogroup labels C1, C2, C3, C4, and C5 as they are used in [9]. 31 of the 144 typed individuals carried Y chromosomes belonging to one of the indigenous haplogroups. Among these individuals, five from haplogroup C, six from haplogroup K^∗^, and two from haplogroup M were re-contacted and agreed to further studies, so their genomes were sequenced to high coverage using the Illumina HiSeq platform (Table 1). Consent was provided to study the history of the uniparental chromosomes, and reads mapping to the Y chromosome were identified. These form the basis for the current study. Comparative data on the sequences of Y chromosomes from other continents were obtained from phase 3 of the 1000 Genomes Project [10], comprising 1,244 samples from 26 populations falling into a wide range of haplogroups, as well as from 12 samples from Papua New Guinea [11] which fall into the haplogroups C, M, and K^∗^ as expected [12, 13].

### Construction of a Y Chromosome Phylogeny

**Figure 1:**
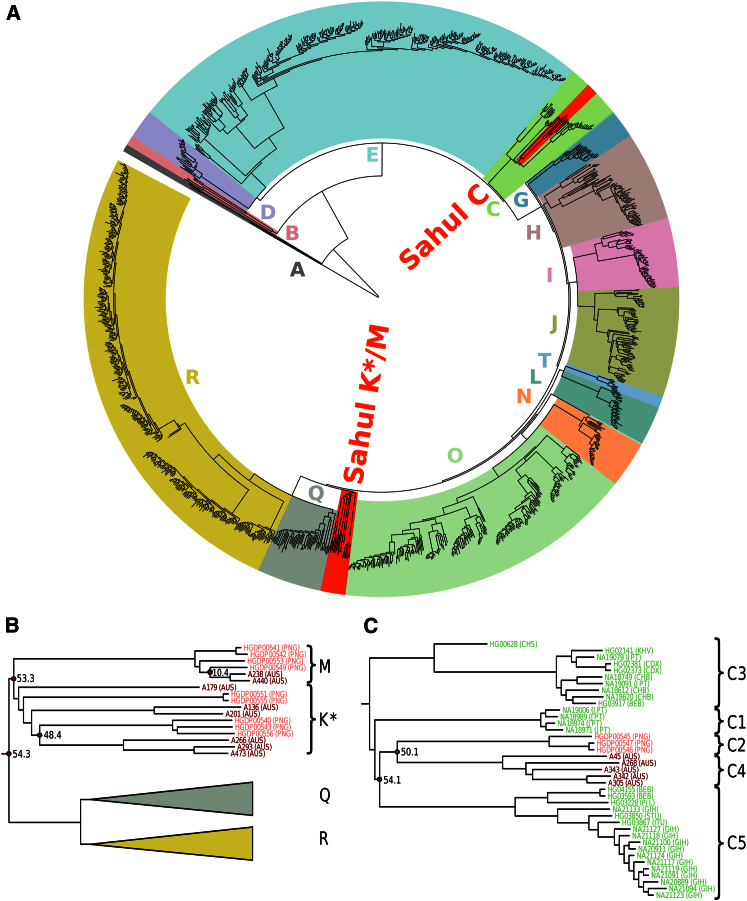
Phylogenetic History of Aboriginal Australian Y Chromosomes (A) A maximum-likelihood phylogeny was inferred from the Y chromosome data of 1,269 males from worldwide populations, including Aboriginal Australians, using RAxML [14]. High-level haplogroups are colored and labeled along the tree. The two clades that contain the Y chromosomes indigenous to the continent of Sahul (from the Aboriginal Australian and Papuan samples) are indicated in bright red. (B) The phylogeny of Y chromosomes in haplogroups K^∗^ and M. This detailed view of a part of the larger tree displayed in (A) focuses on chromosomes in haplogroups K^∗^ and M. Haplogroups Q and R, which are the closest relatives to K^∗^ and M in the phylogeny, are represented schematically because they contain very large numbers of samples. Aboriginal Australian and Papuan samples are colored in two different shades of red for easier visual separation. Sample names and population origins are displayed at branch tips (AUS, Aboriginal Australian; PNG, Papua New Guinean). Divergence times in units of thousands of years are indicated on key nodes that correspond to divergences between groups of samples from different populations or haplogroups. (C) The phylogeny of Y chromosomes in haplogroup C. Sample names and population origins are displayed at branch tips (AUS, Aboriginal Australian; PNG, Papua New Guinean; CHS, Southern Han Chinese in China; KHV, Kinh in Ho Chi Minh City, Vietnam; JPT, Japanese in Tokyo, Japan; CDX, Chinese Dai in Xishuangbanna, China; CHB, Han Chinese in Bejing, China; BEB, Bengali in Bangladesh; PJL, Punjabi in Lahore, Pakistan; GIH, Gujarati Indian in Houston, Texas; STU, Sri Lankan Tamil in the UK; ITU, Indian Telugu in the UK). We note that due to factors associated with missing data arising from the low sequencing coverage of the 1000 Genomes samples, the branch lengths displayed here are not strictly proportional to time. See also Table S1.

We used the sequence data to infer a maximum-likelihood phylogenetic tree for the 1,269 Y chromosomes (Figure 1A) (see the Experimental Procedures and Supplemental Experimental Procedures). The overall topology of the tree recapitulates the known Y chromosome phylogeny. In agreement with the prior haplogroup assignments, the Aboriginal Australian and Papuan Y chromosomes fall into two distinct monophyletic clades within the C and K^∗^/M haplogroups. Both of these clades received high bootstrap support (100% for the haplogroup C samples and 97% for the haplogroup K^∗^/M samples). The shared phylogenetic history of Aboriginal Australian and Papuan Y chromosomes is consistent with the common origin of these populations as previously inferred from genome-wide data [4, 15, 16, 17].

### Divergence Times between Aboriginal Australian and Other Y Chromosomes

The phylogenetic tree reveals deep divergences between Y chromosomes indigenous to Sahul, the ancient continent that included both Australia and New Guinea, and those from all other populations (Figures 1B and 1C). Complete sequence data allow direct and accurate inference of the timing of these divergences. Applying a point mutation rate of 0.76 × 10^−9^ per site per year inferred from the number of missing mutations on the Y chromosome of a ∼45-ky-old radiocarbon-dated Eurasian sample [18], we infer a divergence time of 54.3 ky (95% confidence interval [CI]: 48.0–61.6 ky) between K^∗^/M chromosomes in Sahul and their closest relatives in the R and Q haplogroups (Figure 1B), and a divergence time of 54.1 KY (95% CI: 47.8–61.4 ky) between Sahul C chromosomes and their closest relatives in the C5 haplogroup (Figure 1C), a distinction noted previously on the basis of a single SNP, M347 [9]. These dates are consistent with the archeological record documenting human occupation in Australia by ∼47 kya [2] and with genome-wide analyses that have found an early divergence between the ancestors of Eurasian populations and the ancestors of Aboriginal Australians and Papuans [15]. They thus provide no evidence for any later Y chromosome gene flow into Australia between the early separation and the beginning of recent European colonization. Specifically, these results refute earlier findings based on short tandem repeat (STR) variation that Aboriginal Australian Y chromosomes in the C haplogroup descend from populations in southern India and Sri Lanka 1.3–13.3 kya [5]. Although the closest chromosomes to the Aboriginal Australian Cs in our phylogeny are found in South Asian populations, the deep divergence time and the fact that the Aboriginal Australian Cs share a more recent common ancestor with Papuan Cs show that this is not the result of recent genetic contact. The CIs reported above take into account the uncertainty of the Y chromosome point mutation rate, but not necessarily other possible sources of technical uncertainty (such as read alignment and genotype calling). We tested whether accounting for such additional uncertainty could affect the conclusion of a deep divergence between Aboriginal Australian and South Asian C chromosomes by re-estimating this divergence time from 100 bootstrap samples of sites from the full ∼10 million analyzed Y chromosome sites. The 95% CI for these estimates was 50.9–58.1 kya, and very conservative application of the mutation rate uncertainty multiplicatively to the bootstrap estimates gives a combined CI of 44.9–65.9 kya. Technical uncertainty is thus not large enough to affect our overall conclusion. The disparity between our findings and the earlier report can be attributed to improvements in technology, as none of the methods previously used to study the history of the paternal lineage offered the level of phylogenetic or dating precision afforded by complete Y chromosome sequencing. Redd et al. employed ten widely used Y STRs (three simple trinucleotides, three simple tetranucleotides, one of which was bilocal, and four complex tetranucleotides), applying the same fast genealogical mutation rate of 2.08 × 10^−3^ per STR per 25 years to all of them [5]. It has been shown that Y STRs tend to massively under-estimate ancient divergence times [19], perhaps because of a combination of the fast mutation rate assumed, saturation of STR distances, and in this case the short generation time used.

Although the shared origin of Aboriginal Australians and Papuans is clearly established, and now also supported by the Y chromosome phylogeny presented here, little is known about the history of population separation and gene flow between these groups within Sahul. We observe deep divergences between Aboriginal Australian and Papuan Y chromosomes within the C (50.1 ky; 95% CI: 44.3–56.9 ky) (Figure 1C) and the K^∗^ (48.4 ky; 95% CI: 42.8–54.9 ky) (Figure 1B) haplogroups. Although this would be consistent with an early split between the populations, we note that our limited sample size makes it very unlikely that we have observed the most recent divergences, and we therefore cannot rule out more recent split times. Within the M haplogroup, which is found at high frequencies in Papua New Guinea and Melanesia [20] but in less than 1% of Aboriginal Australian males [6], we find a divergence time of 10.4 ky (95% CI: 9.2–11.9 ky) (Figure 1B). Although this coincides approximately with the post-glacial geographical separation of Australia and New Guinea after the rise of the sea level ∼6–8 kya [21], the fact that the two Aboriginal Australian males who carry the haplogroup M chromosomes trace their paternal ancestry to the Torres Strait Islands (Table 1) makes it more likely that these are very recent introductions into the mainland Australian gene pool. A larger number of geographically diverse Y chromosomes from the different haplogroups indigenous to Sahul would be needed in order to learn more about population relationships within the continent.

### Implications for the Peopling of Australia

Y haplogroups from Australia and Papua New Guinea were estimated to diverge from the nearest non-Sahul lineages ∼54 kya, and divergences within Sahul-specific lineages date to ∼48–53 kya. We note that these times post-date the Mount Toba eruption ∼74 kya [22], supporting a model of the initial peopling of this region by modern humans long after this event. The divergence times are close to, but earlier than, the current conservative archaeological date for entry into Sahul, 47 kya [2]. However, the uncertainty in the lineage divergence estimates and the possibility that earlier archaeological sites may be detected make it impossible to determine whether the initial divergence within the Sahul-specific lineages occurred before or after entry into Sahul. The current evidence is consistent with a simple model of a single entry and subsequent rapid lineage divergence.

Around the mid-Holocene (∼4–6 kya), small stone tools began to be used extensively in Australia [3], the Pama-Nyungan language family spread over most of the mainland [23], and the first archaeological evidence for the dingo appeared [3]. Genetic patterns proposed as indications of gene flow into Australia from South Asia were dated to approximately the same period. One parsimonious interpretation of these diverse findings could be that they were all linked, and thus that there was a substantial and influential population influx at this time.

We have taken advantage of improvements of sequencing technology [10] and calibration of the molecular clock [18] to re-examine the claim for male gene flow revealed by Y chromosome relationships [5]. Our sample of 13 Aboriginal Australian Y chromosomes is small, but it includes the relevant haplogroups and conclusively refutes the original basis for this claim. Although this does not demonstrate the absence of any Holocene gene flow or non-genetic influences from South Asia at this time, and the appearance of the dingo remains as strong evidence for external contacts, the evidence overall is consistent with a complete lack of gene flow and indigenous origins for the technological and linguistic changes.

Australia and Papua New Guinea are currently separated only by the 150-km-wide Torres Strait, in which lie many islands. Gene flow across this Strait is both geographically plausible and demonstrated by our data, although we cannot determine when within the last 10 ky it occurred. The analytical techniques now available, applied to larger genetic datasets, including ancient DNA, have the potential to address such questions and provide more detailed insights into the human history of Sahul.

## Experimental Procedures

This study received ethical approval from the La Trobe University Human Ethics Committee, Melbourne, Australia (HEC 05/94, April 11, 2006; amended April 18, 2012, June 26, 2012) and The Wellcome Trust Sanger Institute Human Materials and Data Management Committee, Hinxton, UK (12/055). Conclusions from the study have been returned to the participants. We sequenced the whole genomes of 13 Aboriginal Australian males to high coverage on the Illumina HiSeq platform and then analyzed only the reads mapping to the Y chromosome. We used FreeBayes to determine the genotypes of these individuals, along with those of 1,244 males sequenced to low coverage in the 1000 Genomes Project [10] and 12 males from Papua New Guinea sequenced to high coverage [11], at ∼10 million Y chromosome sites accessible by short read sequencing. We then used RAxML [14] to infer a maximum-likelihood phylogeny of all the 1,269 Y chromosomes. We estimated the divergence times between clades in the tree by applying the ρ statistic [24], aggregating data across low-coverage samples where relevant, and converted divergence times to units of years by applying a mutation rate of 0.76 × 10^−9^ per site per year [18]. For more detailed descriptions of the sequence data processing, genotyping and filtering, phylogenetic inference, and dating, see the Supplemental Experimental Procedures. Table S1 provides information on the SNPs called that are phylogenetically informative for the branches of the Y chromosome phylogeny specific to Aboriginal Australians and Papuans (see the Supplemental Experimental Procedures for a description of this table).

## Author Contributions

Project design was carried out by Y.X., R.J.M., and C.T.-S.; community engagement, ethics, and sampling by S.W., L. Wilcox, R.A.H.v.O., P.M., L. Williams, and R.J.M.; data generation, processing, and analysis by A.B., N.N., Y.C., S.M., M.O.P., Q.A., Y.X., and C.T.-S.; data interpretation by A.B., N.N., S.W., R.A.H.v.O., P.M., L. Williams, Y.X., R.J.M., and C.T.-S.; and manuscript writing by A.B., Y.X., R.J.M., and C.T.-S.

## Figures and Tables

**Table 1:** Aboriginal Australian Individuals Sampled for This Study

ID	Y Coverage	Haplogroup	Key Variant	Paternal Origin
A45	19.74	C	M130	Uncertain, possibly Normanton, Queensland
A268	13.06	C	M210	Atherton Tablelands, Far North Queensland
A305	18.03	C	M347	The Karryarra group located near Port Hedland, Western Australia
A342	18.06	C	M347	The Karryarra group located near Port Hedland, Western Australia
A343	12.90	C	M347	Northwest coast, near Broome, Western Australia
A136	12.61	K^∗^	M526	Kuranda, Far North Queensland
A179	18.85	K^∗^	M526	Gunganji tribe, Yarrabah, near Cairns, Far North Queensland
A201	12.19	K^∗^	M526	Uncertain, but states father’s people from South East Queensland
A266	19.07	K^∗^	M526	Gunganji tribe, Yarrabah, near Cairns, Far North Queensland
A293	12.77	K^∗^	P308	Pilbara, Western Australia
A473	13.73	K^∗^	P308	Mount Isa region, Central Queensland
A238	16.42	M	M186	Mer (Murray Island), Torres Strait, Far North Queensland
A440	15.29	M	M186	Mer (Murray Island), Torres Strait, Far North Queensland

“Y coverage” refers to the average depth of sequencing coverage on the Y chromosome. We note that the geographic information on the origin of the paternal line is sometimes uncertain and, due to the widespread movement of Aboriginal people after European colonization, might not reflect deeper geographic origins.

## Accession Numbers

Y chromosome sequence data from the 13 Aboriginal Australians are available for studies of population history under managed access through two separate study accession numbers at the European Genome-phenome Archive (EGA): EGAS00001000315 and EGAS00001000718, both with EGA DAC accession number EGAC00001000205 and EGA policy accession number EGAP00001000210. The correspondence between the sample IDs used in this manuscript and the sample accession numbers is as follows: A45, EGAN00001072788; A136, EGAN00001196788; A179, EGAN00001072787; A201, EGAN00001072789; A238, EGAN00001089015; A266, EGAN00001089016; A268, EGAN00001192719; A293, EGAN00001192720; A305, EGAN00001089017; A342, EGAN00001088616; A343, EGAN00001192721; A440, EGAN00001196789; and A473, EGAN00001196790. Details of SNPs called within the Sahul-specific branches are provided in Table S1. We request that L. Williams (geraniumgroup@gmail.com), R.J.M., and C.T.-S. be consulted before any commercial use is made of novel SNPs within this table.

## Supplemental Information

Document S1. Supplemental Experimental Procedures

Table S1. Novel Y-SNPs within Haplogroups C and K∗/M, Related to Figure 1 Column A: chromosomal position in build GRCh37. B: Reference allele. C: Alternative allele. D: ISOGG information (January 18th, 2014 version (v9.05), http://isogg.org/), where available (. = no information). E-AC: allele called in each of the 25 Aboriginal Australian and Papua New Guinean samples. These are the SNPs that define the branches highlighted in red/dark red in Figures 1A–1C. For more information, see the Supplemental Experimental Procedures.

Document S2. Article plus Supplemental Information
