# Supplementary material for: Deep Roots for Aboriginal Australian Y Chromosomes
Source: Curr Biol. 2016 Mar 21;26(6):809–13. doi: 10.1016/j.cub.2016.01.028 (PMC4819516; doi:10.1016/j.cub.2016.01.028)
Supplement: Document S1. Supplemental Experimental Procedures [file mmc1.pdf]

Current Biology, Volume *26*

Supplemental Information

Deep Roots for Aboriginal Australian Y Chromosomes

Anders Bergström, Nano Nagle, Yuan Chen, Shane McCarthy, Martin O. Pollard, Qasim Ayub, Stephen Wilcox, Leah Wilcox, Roland A.H. van Oorschot, Peter McAllister, Lesley Williams, Yali Xue, R. John Mitchell, and Chris Tyler-Smith

Table S1. Novel Y-SNPs within haplogroups C and K*/M. Column A: chromosomal position in build GRCh37. B: Reference allele. C: Alternative allele. D: ISOGG information (January 18th, 2014 version (v9.05), http://isogg.org/), where available (. = no information). E-AC: allele called in each of the 25 Aboriginal Australian and Papua New Guinean samples. These are the SNPs that define the branches highlighted in red/dark red in Figure 1A, 1B and 1C. For more information see Supplemental Experimental Procedures.

This table is provided as an Excel file.

SUPPLEMENTAL EXPERIMENTAL PROCEDURES

Samples

The 13 males included in this study are drawn from a sample collected as part of a larger study of worldwide genomic variation, the Genographic Project, conducted between 2005 and 2013. This project focused on variation within mitochondrial DNA and the Y chromosome. In Australia, the project attempted as wide a geographic coverage of Aboriginal Australians as possible.

We contacted Aboriginal elders in each region to help publicise the Genographic Project and held local meetings to which they were invited. All who identified as Aboriginal were invited to participate in the study, even if they knew they had no direct maternal or paternal line of Aboriginal descent. Aboriginality is a culturally based affiliation and not defined by a person's genetic make-up. DNA was extracted from a saliva sample collected using the Oragene collection kit (http://www.dnagenotek.com/ROW/products/OG500.html).

Following genotyping within the Genographic Project and feedback of results to participants, we attempted to re-contact all haplogroup C, K* and M males to seek their permission for full sequencing of their Y chromosome, and received this permission from 13. Where required, a second saliva sample was collected and DNA extracted as described [S1].

The present study received ethical approval from the La Trobe University Human Ethics Committee, Melbourne, Australia (HEC 05/94, 11th April 2006; amended 18th April 2012, 26th June 2012) and The Wellcome Trust Sanger Institute Human Materials and Data Management Committee, Hinxton, UK (12/055). Conclusions from the study have been returned to the participants.

DNA sequencing, data processing and genotype calling

A single sequencing library was constructed per sample and each library was sequenced across multiple lanes on the Illumina HiSeq platform (read length 100 base-pairs, target fragment length 350 base-pairs).

The sequence reads were mapped to the hs37d5 version of the human reference genome using bwa aln v0.5.9 with the argument "-q 15". Duplicate reads were marked using Picard MarkDuplicates v1.06. The consent obtained from the sample donors only allowed study of the Y chromosome and mitochondrial DNA, and therefore only read pairs mapping properly to these parts of the genome were accessed for further analysis. Mitochondrial DNA results will be reported elsewhere. GATK v.3.3 IndelRealigner was used to perform local realignment around known indels on the Y chromosome (the "Mills-Devine" indel set and the "20101123" 1000 Genomes Phase 2 low coverage indel set, both obtained from the 1000 Genomes Project), with the argument "–LOD 0.4". Base Alignment Qualities (BAQ) were computed using samtools [S2]. These steps were performed so as to largely match the processing applied to the low coverage read alignments in 1000 Genomes Phase 3. Depth of coverage along the chromosome was calculated using samtools [S2].

Y chromosome read alignments for all 1244 male samples in Phase 3 were obtained from the 1000 Genomes Project [S3] (mean Y chromosome coverage = 3.81). Y chromosome read alignments for 12 male samples from Papua New Guinea sequenced to high-coverage were obtained from [S4] (mean Y chromosome coverage = 9.91).

Genotypes were called jointly across all 1269 samples using FreeBayes v0.9.18 [S5] with the arguments "—ploidy 1" to accommodate the haploid state of the Y chromosome and "—report-monomorphic" to obtain genotype calls also at sites without any evidence for polymorphism. Calling was restricted to the regions of the Y chromosome deemed suitable for Illumina short read sequencing [S6], totaling 10,445,993 base-pairs. Furthermore, sites on which the data fulfilled any of the following criteria were excluded:

1) A depth of coverage of reads with mapping quality greater than or equal to 1 in the top or bottom 1% of sites (corresponding to <2401 or >6064 such reads across all samples)

2) A ratio greater than 0.1 of the number of reads with mapping quality 0 to the total number of reads
3) A fraction greater than 0.3 of the number of samples with missing genotype in the unfiltered genotype calls
4) A number of samples greater than 200 having more than 1 read not supporting the called genotype at the site

These filters are similar to those used for the Y chromosome in the 1000 Genomes Project [S3]. The sample genotypes at these sites were then recalled by setting the genotype to the allele with the highest number of supporting reads, or to missing if no allele was supported by at least 2 reads, if more than one allele was supported by more than 1 read or if the fraction of reads supporting the majority allele was lower than 0.75. Multiple alleles at a site were all retained and sites containing possible indel alleles were not removed. Multi-nucleotide variants were decomposed to their constituent SNPs and/or indels using the tool vcfallelicprimitives from the vcflib library (https://github.com/ekg/vcflib). 9,891,532 sites with data remained for analyses. Additional site filters were applied specifically for the purpose of phylogenetic tree inference, but not for dating of split times (see below).

Phylogenetic inference and dating

A maximum likelihood phylogeny of all 1269 Y chromosomes was inferred using RAxML v8.1.15 [S7]. To reduce the computational burden, only SNP sites with a QUAL score greater than or equal to 1 were used as input for this inference, totalling 53,813 sites. RAxML was run with the ASC_GTRGAMMA model of nucleotide substitution and the "stamatakis" correction for ascertainment bias, directly specifying to the program the number of invariable sites. Statistical support for each clade in the tree was assessed from 100 bootstrap replicates. Trees were visualized using the Interactive Tree Of Life [S8] and the FigTree software (http://tree.bio.ed.ac.uk/software/figtree/) and manually rooted at the branch leading to samples in the A0 haplogroup.

The genotypes of the 12 samples in the A and B haplogroups, which are the earliest branching lineages in the Y-chromosomal phylogeny and an outgroup to all the lineages that form the focus of this study, were used to define the ancestral nucleotide state at all sites. Pooling of data across multiple samples in this fashion allows the ancestral state to be accurately inferred at a larger number of sites, as the low sequencing coverage of the 1000 Genomes samples precludes genotype calling at many sites for any given single sample. If there was just one allele present among the called genotypes of these 12 samples at a site, this allele was set as the ancestral nucleotide, while if there were multiple alleles present the ancestral state was not defined. This allowed the ancestral state to be called at all except 4825 sites (0.049%).

The ages of internal nodes in the inferred phylogenetic tree were estimated directly using the ρ statistic [S9] on all called sites. Given a branch between a first sample and an internal node corresponding to the common ancestor with a second sample, the length of the branch was estimated by dividing the number of derived mutations present in the first sample and not the second by the total number of sites with ancestral state in both samples. If either sample had missing data or an indel genotype, the site was ignored. If multiple samples were available in a given clade of the tree, the per-sample divergence estimates were averaged across them. When estimating divergence from a node for which only low coverage 1000 Genomes samples were available for comparison, data were pooled across multiple samples in the way described above for the determination of ancestral states, and the divergence was calculated against the resulting single consensus genotype. Specifically, for dating the divergence of Aboriginal Australian and Papuan haplogroup C chromosomes, data were pooled across the 17 samples in the C5 haplogroup, and for dating the divergence of Aboriginal Australian and Papuan haplogroup K* and M chromosomes data were pooled across 20 randomly selected samples from the R and Q haplogroups.

We converted divergence times in units of mutations per basepair to units of years by applying a point mutation rate of 0.76×10^{-9} mutations per site per year. This rate was inferred from the relative deficiency of mutations on the Y chromosome relative to modern humans of an ancient human sample found near Ust'-Ishim in western Siberia, radiocarbon-dated to have lived ~45 KYA [S10]. This rate is similar to, but slightly lower than, that inferred from multi-generational genealogies of Icelandic males [S11] (~0.88×10^{-9} mutations per site per year), but we reason that the integration over generation times in diverse human populations over 45 KY inherent in the Ust'-Ishim rate estimate is more appropriate for the purposes of the present study. We also note that our main conclusions would not be affected by applying a slightly different mutation rate. We report 95% confidence intervals on the divergence times corresponding to the uncertainty of the Ust'-Ishim mutation rate (0.67×10^{-9} to 0.86×10^{-9}).

Details of novel SNPs and phylogenetic inferences within haplogroups C and K*/M

Our work provides new insights into the early and Sahul-specific differentiation within haplogroups C and K*/M, and we provide information on high-quality discovered SNPs that are informative for these branches in an accessible form in Table S1. This table lists the chromosomal position of each SNP in the GRCh37/hg19 coordinate system, the reference genome allele and the alternative allele (we note that for all these SNPs we have confirmed that the reference allele is the ancestral allele), any existing marker labels present in the January 18th, 2014 version (v9.05) of the ISOGG database (http://isogg.org/) and the genotype of all 25 Aboriginal Australian and Papua New Guinean samples included in this study (where "0" denotes the reference allele and "1" denotes the alternative allele). Of the variants listed in Table 1, M186 is absent from Table S1 because it is a deletion, while M130 and M526 are absent because they lie deeper in the phylogeny than the lineages included in Table S1.

Haplogroups C2 and C4 together form a monophyletic group and in our data are distinguished from the common ancestor with C5 by 25 high-confidence SNPs; similarly, Aboriginal Australian C4 and Papua New Guinean C2 samples form two distinct monophyletic clusters. Within the K*/M monophyletic haplogroup, which is distinct from QR, M forms the first branch, with the sampled Aboriginal Australian and Papua New Guinean chromosomes sharing a common ancestor within the last ~22 KY. The remaining K* chromosomes analysed fall into five deep clades (>48 KY), three Aboriginal Australian and two Papua New Guinean. This phylogeny is consistent with the most detailed previous phylogenetic analysis of haplogroup K [S12], resolving the multifurcation within the lineage there designated K2b1 (here K*/M). Since it seems likely that further complexity may be revealed in this part of the phylogeny by additional sequencing studies in the near future, we have refrained from assigning alphanumeric labels to the clades, and suggest that if necessary they are referred to using the haplogroup and first variant listed in Table S1, e.g. K*(GRCh37- 2,658,341-T) for the lineage defined by HGDP00540, HGDP00543 and HGDP00556.

SUPPLEMENTAL REFERENCES

S1. Nagle, N., Ballantyne, K.N., van Oven, M., Tyler-Smith, C., Xue, Y., Taylor, D., Wilcox, L., Turkalov, R., van Oorschot, R.A.H., McAllister, P., et al. (2015). Antiquity and diversity of Aboriginal Australian Y-chromosomes. Am. J. Phys. Anthropol. *online*.

S2. Li, H., Handsaker, B., Wysoker, A., Fennell, T., Ruan, J., Homer, N., Marth, G., Abecasis, G., Durbin, R., and 1000 Genome Project Data Processing Subgroup. (2009). The Sequence Alignment/Map format and SAMtools. Bioinformatics *25*, 2078-2079.

S3. The 1000 Genomes Project Consortium (2015). A global reference for human genetic variation. Nature *526*, 68-74.

S4. Raghavan, M., Steinrucken, M., Harris, K., Schiffels, S., Rasmussen, S., DeGiorgio, M., Albrechtsen, A., Valdiosera, C., Avila-Arcos, M.C., Malaspinas, A.S., et al. (2015). Genomic evidence for the Pleistocene and recent population history of Native Americans. Science *349*, aab3884.

S5. Garrison, E., and Marth, G. (2012). Haplotype-based variant detection from short-read sequencing. arXiv preprint, arXiv:1207.3907 [q-bio.GN].

S6. Poznik, G.D., Henn, B.M., Yee, M.C., Sliwerska, E., Euskirchen, G.M., Lin, A.A., Snyder, M., Quintana-Murci, L., Kidd, J.M., Underhill, P.A., et al. (2013). Sequencing Y chromosomes resolves discrepancy in time to common ancestor of males versus females. Science *341*, 562-565.

S7. Stamatakis, A. (2014). RAxML version 8: a tool for phylogenetic analysis and post-analysis of large phylogenies. Bioinformatics *30*, 1312-1313.

S8. Letunic, I., and Bork, P. (2011). Interactive Tree Of Life v2: online annotation and display of phylogenetic trees made easy. Nucleic Acids Res *39*, W475-478.

S9. Forster, P., Harding, R., Torroni, A., and Bandelt, H.J. (1996). Origin and evolution of Native American mtDNA variation: a reappraisal. Am J Hum Genet *59*, 935-945.

S10. Fu, Q., Li, H., Moorjani, P., Jay, F., Slepchenko, S.M., Bondarev, A.A., Johnson, P.L., Aximu-Petri, A., Prufer, K., de Filippo, C., et al. (2014). Genome sequence of a 45,000-year-old modern human from western Siberia. Nature *514*, 445-449.

S11. Helgason, A., Einarsson, A.W., Guethmundsdottir, V.B., Sigurethsson, A., Gunnarsdottir, E.D., Jagadeesan, A., Ebenesersdottir, S.S., Kong, A., and Stefansson, K. (2015). The Y-chromosome point mutation rate in humans. Nat Genet *47*, 453-457.

S12. Karafet, T.M., Mendez, F.L., Sudoyo, H., Lansing, J.S., and Hammer, M.F. (2015). Improved phylogenetic resolution and rapid diversification of Y-chromosome haplogroup K-M526 in Southeast Asia. Eur J Hum Genet *23*, 369-373.
